# Chikungunya Awareness and Related Knowledge, Attitudes, and Practices Among Nursing Students at the University of Palermo, Italy: A Cross-Sectional Study of Associated Sociodemographic Factors

**DOI:** 10.3390/epidemiologia7050141

**Published:** 2026-10-09

**Authors:** Omar Enzo Santangelo, Sandro Provenzano, Luana Maria Amato, Simona Fanuli, Anna Sole Pizzamiglio, Carlotta Vella, Giuseppina Pesco, Giuseppe D’Anna, Alberto Firenze, Antonio Terranova

**Affiliations:** 1 Territorial Health Care and Social Agency of Lodi, Azienda Socio Sanitaria Territoriale di Lodi, 26900 Lodi, Italy; annasole.pizzamiglio@asst-lodi.it (A.S.P.); carlotta.vella@asst-lodi.it (C.V.); 2 Hospital Management, Local Health Unit of Trapani, Azienda Sanitaria Provinciale di Trapani, 91100 Trapani, Italy; provenzanosandro@hotmail.it; 3 Villa Sofia-Cervello United Hospitals, 90146 Palermo, Italy; luanamariaamato@gmail.com (L.M.A.); simonafanuli@hotmail.it (S.F.); 4 Integrated Medical Activities Department, University Hospital “P. Giaccone” Hospital, 90133 Palermo, Italy; giuseppina.pesco@policlinico.pa.it; 5 Department of Health Promotion, Maternal-Infant, Internal Medicine and Specialization of Excellence ‘G. D’Alessandro’, University of Palermo, 90127 Palermo, Italy; giuseppe.danna@unipa.it (G.D.); antonio.terranova01@unipa.it (A.T.); 6 General Management, Local Health Unit of Palermo, Azienda Sanitaria Provinciale di Palermo, 90141 Palermo, Italy; alberto.firenze@unipa.it

**Keywords:** chikungunya, Italy, vector control, health education, nursing students

## Abstract

Introduction: Chikungunya is an emerging mosquito-borne viral disease that has become a growing public health concern worldwide. Despite documented autochthonous outbreaks in Italy and the increasing spread of *Aedes albopictus* mosquitoes across Europe, little is known about awareness of chikungunya among future healthcare professionals. This study aimed to assess awareness of chikungunya and evaluate related knowledge, attitudes, and practices (KAPs) among nursing students at the University of Palermo, Italy. Methods: A cross-sectional study was conducted in May 2026 among undergraduate nursing students enrolled at the University of Palermo. Data were collected through an anonymous self-administered online questionnaire investigating sociodemographic characteristics, awareness of chikungunya, sources of information, and KAP related to the disease. Associations between awareness and participant characteristics were assessed using chi-square tests and prevalence ratio analyses. Results: A total of 894 students participated (mean age: 22.33 ± 4.53 years; 72.3% female). Overall, only 22 students (2.46%) reported having previously heard of chikungunya. In a multivariable exploratory analysis, third-year students had a higher prevalence of previous awareness compared with first-year students (PR = 3.38; 95% CI: 1.10–10.36), whereas no statistically significant association was observed for age (PR = 1.07; 95% CI: 1.00–1.15). Mass media represented the main source of information (63.6%), followed by university lectures (22.7%). Among participants who reported previous awareness, responses suggested relatively good recognition of major clinical manifestations, although important gaps were observed regarding vector recognition and transmission pathways. Attitudes toward prevention were predominantly positive, and most participants reported adopting recommended mosquito-control practices. Conclusions: Awareness of chikungunya among nursing students was extremely low. Strengthening educational interventions on emerging vector-borne diseases within nursing curricula may improve preparedness, risk communication, and future public health responses.

## 1. Introduction

Chikungunya is an emerging mosquito-borne disease caused by the chikungunya virus (CHIKV), an arthropod-borne alphavirus belonging to the family Togaviridae. The virus is transmitted primarily through the bites of infected *Aedes aegypti* and *Aedes albopictus* mosquitoes and typically causes an acute febrile illness characterized by severe polyarthralgia, headache, myalgia, skin rash, and fatigue [1,2]. Although the disease is associated with low mortality, it may result in substantial morbidity due to the persistence of musculoskeletal manifestations, particularly arthralgia and arthritis, which can last for months or years after the acute infection and significantly impair quality of life [2,3,4]. Over the past two decades, chikungunya has re-emerged as a major global public health concern. Following large outbreaks in Africa, Asia, and islands of the Indian Ocean, CHIKV has expanded geographically and now circulates in more than 100 countries and territories worldwide [2,5]. This expansion has been driven by multiple factors, including globalization, increasing international travel, rapid urbanization, environmental modifications, and the widespread dissemination of competent mosquito vectors [5,6]. Consequently, chikungunya is now recognized as one of the most important emerging arboviral diseases, posing significant challenges for surveillance systems and public health authorities worldwide [5]. The epidemiological situation in Europe has changed considerably in recent years due to the successful establishment of *Aedes albopictus*, commonly known as the Asian tiger mosquito. This invasive species, first detected in Italy in the early 1990s, has progressively spread throughout southern and central Europe and has demonstrated a remarkable capacity to adapt to temperate climates and urban environments [7]. Unlike many tropical mosquito species, *A. albopictus* can survive in a wide range of environmental conditions, increasing the likelihood of local transmission of arboviruses following the introduction of infected travelers returning from endemic regions [7,8]. Italy represents a particularly relevant setting for the study of chikungunya because it was the first European country to report documented autochthonous transmission of CHIKV. In 2007, an outbreak in the Emilia-Romagna region resulted in more than 200 locally acquired cases and provided clear evidence that *A. albopictus* was capable of sustaining viral transmission in a temperate European environment [9]. A decade later, a second major outbreak occurred in the Lazio and Calabria regions, resulting in hundreds of confirmed and probable cases and reaffirming the vulnerability of the Italian population to emerging arboviral threats [10,11]. More recently, Italy experienced further autochthonous chikungunya transmission in 2025. According to the national arboviral surveillance system coordinated by the Italian National Institute of Health (Istituto Superiore di Sanità, ISS), 472 confirmed chikungunya cases were reported in Italy in 2025, including 384 autochthonous cases and 88 travel-associated cases. The 384 autochthonous cases occurred during six episodes of local transmission in three Italian regions [12]. According to the European Centre for Disease Prevention and Control (ECDC), these autochthonous cases were reported across eight regions and 27 municipalities, with local transmission occurring between July and November 2025 [13]. These recent events further demonstrate that autochthonous chikungunya transmission remains possible in Italy and underscore the importance of surveillance, vector control, early diagnosis, and public awareness. No autochthonous chikungunya transmission was reported in Palermo during the 2025 events [12,13]. However, the absence of reported local transmission in Palermo should not be interpreted as an absence of the competent mosquito vector. *Aedes albopictus* is established throughout Italy, and the most recent ECDC/European Food Safety Authority (EFSA) distribution assessment classified the species as established in 107 of 107 Italian administrative units assessed in April 2026 [14]. Therefore, the epidemiological context of Sicily, including Palermo, should be considered in light of the established presence of a competent vector and the potential introduction of CHIKV through infected travelers, even in the absence of documented local transmission in 2025. This context further supports the importance of maintaining awareness and preparedness regarding emerging arboviral diseases among healthcare professionals and students.

These outbreaks highlighted the importance of integrated surveillance systems, vector control measures, early diagnosis, and public awareness in preventing and mitigating disease transmission [10,11]. Recent evidence suggests that the risk of chikungunya outbreaks in Europe may increase further in the coming decades. Climate change has emerged as a critical driver influencing the distribution, abundance, seasonal activity, and vector competence of *Aedes* mosquitoes [15,16,17]. Rising temperatures may accelerate mosquito development, shorten viral incubation periods, and extend the duration of transmission seasons, thereby creating increasingly favorable conditions for arboviral circulation in Mediterranean countries [15,16]. Epidemiological and modeling studies indicate that southern European regions, including Italy, are likely to remain among the areas at highest risk for future chikungunya emergence because of the widespread presence of *A. albopictus* and the progressive suitability of local climatic conditions [16,17]. Public awareness and community participation are fundamental components of effective chikungunya prevention and control strategies. Since no widely implemented specific antiviral treatment exists and vector control remains the cornerstone of prevention, individual and community behaviors play a crucial role in reducing mosquito breeding sites and minimizing human-vector contact [5]. Adequate knowledge regarding disease transmission, symptoms, risk factors, and preventive measures may facilitate the adoption of protective behaviors and encourage timely healthcare-seeking when symptoms occur [18]. Conversely, poor awareness may delay diagnosis, hinder outbreak detection, and reduce compliance with public health recommendations. Healthcare professionals are central to the prevention and management of emerging infectious diseases. Beyond their clinical responsibilities, they contribute to health promotion, patient education, risk communication, and community engagement during public health emergencies. Nursing professionals, in particular, often represent the first point of contact between healthcare services and the general population. Therefore, nursing students should acquire adequate competencies regarding emerging vector-borne diseases during their undergraduate training in order to effectively support future prevention and control efforts [19]. Despite the growing public health relevance of chikungunya in Europe and the documented occurrence of autochthonous transmission in Italy, evidence regarding awareness and understanding of this disease among nursing students remains limited. Most knowledge, attitudes, and practices (KAPs) studies on chikungunya have been conducted in endemic or outbreak-affected settings and have primarily focused on the general population, healthcare workers, or other population groups. In contrast, relatively little is known about awareness and preparedness among nursing students in non-endemic European settings. This represents a relevant knowledge gap because nursing students are future healthcare professionals who may contribute to health education, risk communication, early recognition of suspected cases, and prevention of vector-borne diseases. Assessing their awareness and identifying potential educational gaps may therefore provide useful information for strengthening undergraduate healthcare education and preparedness for emerging arboviral diseases.

Therefore, the present study aimed to assess the level of awareness of chikungunya and to evaluate related knowledge, attitudes, and preventive practices (KAPs) among undergraduate nursing students enrolled at the University of Palermo, Italy. Among students who reported previous awareness of chikungunya, the study additionally aimed to describe the main sources of information and to assess disease-related knowledge, attitudes, and preventive practices. Finally, we investigated the sociodemographic and academic factors associated with previous awareness of chikungunya, with particular consideration of age, gender, country of birth, and year of study. Because the KAP assessment was restricted to participants who reported previous awareness of chikungunya, these analyses were intended to provide a descriptive characterization of knowledge, attitudes, and preventive practices among this subgroup rather than an estimate of KAP in the entire study population.

## 2. Materials and Methods

A cross-sectional study was conducted during the first week of May 2026 among undergraduate nursing students attending the University of Palermo, Palermo, Italy. Data were collected through an anonymous self-administered online questionnaire distributed during scheduled academic lectures. Attendance at lectures is mandatory within the nursing degree program in order to be eligible for final examinations. The questionnaire was created specifically by the authors. Consequently, the questionnaire was administered to all students present in class at the time of data collection, allowing broad participation across different academic years. The study population included undergraduate students enrolled in the nursing degree program who were present during the survey administration period. Participation was voluntary and anonymous. Before questionnaire distribution, students received information regarding the study objectives and were informed that completion of the questionnaire implied consent to participate. No personal identifiers or sensitive identifying information were collected, ensuring participant confidentiality and anonymity throughout the study process.

Data were collected using an anonymous questionnaire specifically developed ad hoc by the authors for the purposes of this exploratory study. The questionnaire was designed to address the specific objectives of the study and included sections on sociodemographic characteristics, previous awareness of chikungunya, sources of information, knowledge, attitudes, and preventive practices. The knowledge items were based on established epidemiological, clinical, transmission, and prevention characteristics of chikungunya, while the attitude and practice items were designed to explore perceptions and self-reported preventive behaviors relevant to mosquito-borne disease prevention. The questionnaire was not adapted from a previously validated instrument and was not subjected to formal psychometric validation or structured pilot testing before the survey. Because the KAP section was administered only to participants who reported previous awareness of chikungunya, these items were completed by 22 participants. Consequently, the KAP component was considered descriptive and exploratory, and no psychometric validation or reliability analysis of the attitude scale was performed. Before participation, respondents were informed about the anonymous nature of the survey and the voluntary participation in the study. The questionnaire consisted of an initial section investigating participants’ sociodemographic characteristics, including age, gender, year of study, and Country of birth. Participants were also asked whether they had ever heard of Chikungunya (CHIK). Respondents who reported previous awareness of Chikungunya completed additional sections exploring sources of information and Knowledge, Attitudes, and Practices (KAPs) related to the disease. The knowledge section included questions regarding the viral etiology of Chikungunya, modes of transmission, mosquito vectors, preventive measures, and the main clinical signs and symptoms of the disease. Responses were collected using dichotomous (yes/no) or multiple-choice response options, including a “don’t know” category where appropriate. The attitudes section comprised 11 statements assessing perceptions of disease severity, preventability, individual susceptibility, community involvement in vector control, and personal responsibility for prevention. Responses were measured using a 5-point Likert scale ranging from 1 (“strongly disagree”) to 5 (“strongly agree”). The practices section included 10 items assessing participants’ adoption of preventive behaviors aimed at reducing mosquito breeding sites and preventing Chikungunya transmission, such as eliminating standing water, proper waste management, covering water storage containers, and using personal protective measures against mosquito bites. Participants who reported having previously heard of Chikungunya were also asked to indicate one or more sources of information about the disease, including mass media, university lectures, peers/colleagues, and other health-related sources. Categorical variables were summarized as frequencies and percentages, whereas continuous variables were described using means and standard deviations (SDs). The primary outcome of interest was awareness of Chikungunya, defined as a positive response to the question “Have you ever heard of Chikungunya?” (yes/no). Associations between previous awareness of Chikungunya and participants’ sociodemographic and academic characteristics (gender, country of birth, and year of study) were initially assessed using Pearson’s chi-square test, as appropriate. Adjusted Prevalence ratios (PRs) with corresponding 95% confidence intervals (95% CIs) were estimated using multivariable Poisson regression with robust variance. Age was entered as a continuous variable, whereas gender, country of birth, and year of study were entered as categorical variables. Given the small number of participants reporting previous awareness of Chikungunya (n = 22), the multivariable analysis was considered exploratory, and the results were interpreted cautiously. For the analysis of information sources and KAP items, descriptive statistics were calculated and reported as frequencies and percentages. Because the KAP assessment was restricted to the 22 participants who reported previous awareness of Chikungunya, these analyses were descriptive, and no inferential comparisons or regression analyses were performed for KAP variables. For attitude items measured on the Likert scale, mean scores were additionally computed. All statistical tests were two-sided, and a *p*-value < 0.05 was considered statistically significant. Statistical analyses were conducted on the overall study sample (N = 894), whereas KAP analyses were restricted to participants who reported prior awareness of Chikungunya. All statistical analyses were performed using Stata version 13 (StataCorp, College Station, TX, USA). The study was conducted according to the principles of the Declaration of Helsinki. Participation was voluntary, anonymous, and uncompensated. Because no identifiable personal data were collected, confidentiality and privacy were fully guaranteed. The study was approved by the local ethics committee, Palermo 1: Minutes No. 8, meeting of 9 April 2026. During the preparation of this work, the authors used ChatGPT (GPT-5.6 Luna) in order to check the grammar of the manuscript. After using this tool/service, the authors reviewed and edited the content as needed and take full responsibility for the content of the published article.

## 3. Results

### 3.1. Sociodemographic Characteristics and Awareness of Chikungunya

A total of 920 students were enrolled in the degree program at the time of the study, including 350 first-year students, 280 second-year students, and 290 third-year students. Of these, 907 students were present during the survey administration and were invited to participate. Thirteen students declined to provide informed consent and were therefore excluded from the study. The final study sample consisted of 894 participants, corresponding to a participation rate of 98.6% among eligible students present at the time of data collection. A total of 894 participants were included in the study. The majority of respondents were female (72.26%) and born in Italy (98.32%), with a mean age of 22.33 ± 4.53 years. Participants were distributed relatively evenly across academic years, with 38.37% attending the first year, 31.32% the second year, and 30.31% the third year of study.

Overall, awareness of Chikungunya (CHIK) was remarkably low. Only 22 participants (2.46%) reported having previously heard of the disease, whereas the vast majority (97.54%) had never encountered information about CHIK before the survey (Table 1).

### 3.2. Factors Associated with Chikungunya Awareness

The association between sociodemographic characteristics and previous awareness of CHIK is presented in Table 2. No statistically significant differences were observed according to gender (*p* = 0.665) or country of birth (*p* = 0.289). However, awareness significantly differed by year of study (*p* = 0.011), with third-year students reporting higher familiarity with CHIK compared with first- and second-year students (4.80%, 1.17%, and 1.79%, respectively).

Multivariable Poisson regression with robust variance was used to estimate prevalence ratios (PRs) for previous awareness of Chikungunya (Table 3). In the multivariable model, age was not statistically significantly associated with previous awareness (PR = 1.07; 95% CI: 1.00–1.15; *p* = 0.062). Compared with first-year students, third-year students had a higher prevalence of previous awareness (PR = 3.38; 95% CI: 1.10–10.36; *p* = 0.033). No statistically significant associations were observed for gender (PR = 1.17; 95% CI: 0.47–2.93; *p* = 0.730), country of birth (PR = 1.55; 95% CI: 0.11–21.87; *p* = 0.746), or second-year status (PR = 1.45; 95% CI: 0.41–5.21; *p* = 0.565).

### 3.3. Sources of Information on Chikungunya

Among participants who reported previous awareness of CHIK (n = 22), mass media represented the main source of information, reported by 63.64% of respondents. University lectures were the second most frequently cited source (22.73%), whereas peers/colleagues contributed to CHIK awareness in 9.09% of participants. Other less frequently reported sources included yellow fever vaccination centers, previous health-related training, and familiarity with the disease in the country of origin (each 4.55%) (Table 4).

### 3.4. Knowledge, Attitudes, and Preventive Practices Regarding Chikungunya

The knowledge, attitudes, and practices (KAPs) assessment was conducted among the 22 participants who had previously heard of CHIK (Table 5). Among the 22 participants who reported previous awareness of chikungunya, the descriptive assessment of KAP showed heterogeneous patterns of knowledge, attitudes, and preventive practices. Regarding disease etiology, 16 correctly identified CHIK as a viral infection. Most participants correctly recognized that not all mosquitoes transmit CHIK (n = 18) and that mosquitoes breed in stagnant water (n = 17). However, knowledge regarding the vector was limited: only 4 reported being able to recognize the *Aedes* mosquito. Among those who answered this question, most identified that *Aedes* mosquitoes may bite during any period of the day (n = 13), although misconceptions regarding nocturnal biting were reported. Participants showed good recognition of several preventive measures. Most respondents correctly identified the protective role of mosquito repellents (n = 16), mosquito nets (n = 15), and appropriate management of water containers (n = 11). Knowledge of clinical manifestations was generally high, particularly for fever (n = 21), muscle pain (n = 21), headache (n = 19), joint pain (n = 18), and skin rash (n = 17). Among these 22 participants, responses to the attitude items were generally favorable, although substantial variability was observed across individual items. The highest agreement levels were observed for the importance of seeking healthcare when experiencing symptoms (mean score: 4.27), controlling mosquito breeding sites as a prevention strategy (mean score: 4.23), and recognizing individual and community responsibility in CHIK prevention (mean scores ranging from 4.00 to 4.18). Conversely, fewer participants considered CHIK a severe or life-threatening disease (n = 7), and only 7 reported confidence in their own ability to prevent infection. Self-reported preventive practices were frequently reported for several items among the 22 participants. The most commonly adopted behaviors included disposing of water-holding containers (n = 20), regularly cleaning water-filled containers and household drains (n = 20), draining stagnant water areas (n = 19), and changing water in household plant containers weekly (n = 19). Nevertheless, some practices were less frequently reported, including keeping bushes short to reduce mosquito populations (n = 10) and using fans or air conditioning as a preventive measure (n = 6). Overall, despite relatively favorable attitudes and preventive behaviors among the few participants aware of CHIK, the extremely low level of disease awareness in the total sample highlights a substantial gap in knowledge and suggests the need for targeted educational interventions among university students.

## 4. Discussion

This study assessed awareness of chikungunya and related knowledge, attitudes, and practices among nursing students at the University of Palermo, Italy. To our knowledge, few studies have specifically investigated chikungunya awareness and related KAP among nursing students in Italy and, more broadly, in non-endemic European settings. The findings revealed an extremely low level of awareness, with only 2.5% of participants reporting that they had previously heard of the disease. Among those who were aware of chikungunya, knowledge regarding several aspects of the disease was generally moderate, attitudes toward prevention were mostly positive, and reported preventive practices were generally favorable. In the exploratory analysis, third-year students showed a higher prevalence of previous awareness of chikungunya compared with first-year students, whereas age did not reach statistical significance. Given the small number of participants reporting previous awareness (n = 22), these estimates should be interpreted cautiously and should not be considered definitive evidence of independent associations. The most striking finding of the present study was the very low level of chikungunya awareness among nursing students, with only 22 of 894 participants (2.46%) reporting that they had previously heard of the disease. This finding is noteworthy because the study population consisted of future healthcare professionals rather than members of the general population. Previous KAP studies conducted in endemic or outbreak-affected settings have generally reported greater familiarity with chikungunya and other arboviral diseases, although direct comparisons should be made cautiously because of differences in study populations, geographical settings, epidemiological exposure, and questionnaire instruments [20,21,22]. The low awareness observed in our study may partly reflect the non-endemic European context and the limited exposure of students to chikungunya-specific educational content. Although chikungunya is associated with low mortality, its clinical impact may be considerable because acute infection can be followed by prolonged arthralgia, chronic musculoskeletal symptoms, and reduced quality of life, emphasizing the importance of adequate recognition and management of the disease [2,3,4,20]. The low level of awareness observed in our sample may partly reflect the non-endemic context of Italy, where chikungunya is not commonly perceived as an immediate health threat. Previous studies conducted in endemic settings or among populations with direct exposure to outbreaks have reported higher levels of awareness and knowledge regarding chikungunya among university students and healthcare trainees [5,21]. In contrast, students living in non-endemic areas may have limited exposure to specific information on arboviral diseases, despite the increasing risk of local transmission in Europe [10,11,16,17]. The association between academic year and awareness observed in this study suggests that educational exposure may influence knowledge acquisition. Third-year students were significantly more likely to have heard of chikungunya compared with first-year students, indicating that progressive clinical and theoretical training may contribute to greater familiarity with emerging infectious diseases. However, the overall level of awareness remained very low even among senior students, suggesting that arboviral diseases may not yet receive sufficient attention in nursing education programs. Among the 22 participants who reported previous awareness of chikungunya, mass media were the most frequently reported source of information (63.64%), whereas university lectures accounted for 22.73%. This finding suggests that, even among nursing students, awareness of chikungunya may be acquired more frequently through general information channels than through formal academic education. However, this observation should be interpreted cautiously because the source-of-information analysis was based on only 22 participants and therefore cannot be considered representative of the broader nursing student population. The relatively limited contribution of university teaching also supports the potential value of incorporating emerging vector-borne diseases more systematically into healthcare curricula. This finding suggests that information about chikungunya is mainly acquired through informal communication channels rather than structured educational activities. Similar observations have been reported among healthcare workers, where previous studies have shown that knowledge, attitudes, and practices regarding chikungunya are influenced by access to health information, professional exposure, and previous experience with infectious disease prevention activities [21]. These findings support the importance of integrating structured education on emerging vector-borne diseases into healthcare training, as improved knowledge among healthcare professionals may contribute to better risk communication and preventive practices within the community. Although mass media can contribute to rapid dissemination of information during outbreaks, formal education remains essential to ensure accurate knowledge among future healthcare professionals. The KAP findings should be interpreted with particular caution because they were assessed only among the 22 participants who had previously heard of chikungunya. Furthermore, the questionnaire was developed ad hoc and had not undergone formal psychometric validation before administration. Accordingly, the KAP results should be considered exploratory and descriptive rather than representative of the nursing student population as a whole. Nevertheless, several relevant aspects emerged. Participants generally recognized the main clinical manifestations of chikungunya, including fever, joint pain, headache, and muscle pain, which are consistent with the typical presentation of the disease [1,2]. Although recognition of the main clinical manifestations was relatively good, important gaps emerged regarding transmission mechanisms and vector-related prevention. In particular, only 4 of the 22 participants reported being able to recognize the *Aedes* mosquito, while substantial uncertainty was observed regarding person-to-person transmission and other potential transmission pathways. These findings suggest that recognition of the clinical presentation does not necessarily translate into adequate understanding of the mechanisms underlying chikungunya transmission and prevention. This distinction may be particularly relevant to future nursing practice, as nurses may be required not only to recognize suspected cases but also to provide accurate patient education, risk communication, and advice on measures aimed at reducing mosquito exposure and breeding sites. Educational interventions should therefore address the full chain of chikungunya prevention, including vector identification, transmission mechanisms, environmental control, and personal protective measures, rather than focusing exclusively on clinical manifestations. Chikungunya can also be highly debilitating because the characteristic arthralgia may persist for weeks, months, or even years, substantially affecting patients’ functional status and quality of life [2,23]. Although chikungunya is generally associated with low mortality, severe and occasionally fatal outcomes can occur, particularly among older adults, newborns, and individuals with underlying chronic conditions [23,24]. A recent systematic review and meta-analysis including 220,215 patients and 908 fatal cases identified older age, diabetes, hypertension, chronic kidney disease, male sex, and vomiting as factors associated with increased mortality [23]. These findings further emphasize the importance of adequate recognition of suspected cases and knowledge of transmission and prevention mechanisms among healthcare professionals.

Among participants who were already aware of chikungunya, recognition of several characteristic clinical manifestations was relatively high. Fever and muscle pain were identified by 21 participants, while headache and joint pain were recognized by 19 and 18 participants, respectively. In contrast, important gaps emerged regarding vector identification and transmission. Only 4 participants reported being able to recognize the *Aedes* mosquito, while uncertainty was also observed regarding person-to-person transmission and other potential transmission pathways. These findings suggest that familiarity with the clinical presentation of chikungunya does not necessarily translate into adequate knowledge of its vector ecology and transmission mechanisms. Similar gaps in knowledge regarding mosquito vectors and transmission have been reported in studies assessing arboviral KAP among healthcare-related populations [22]. The findings should nevertheless be interpreted in light of the differences between the present study and previous KAP investigations. Studies conducted in areas with endemic or recurrent arboviral transmission may involve participants with greater direct or indirect exposure to chikungunya, whereas students in Italy may have fewer opportunities to encounter disease-specific information outside outbreak periods. Moreover, differences in questionnaire design and in the populations investigated may contribute to variation in reported awareness and knowledge levels. Therefore, the present results should be regarded primarily as preliminary evidence from a specific nursing student population rather than as directly comparable estimates across countries or healthcare professional groups. However, important knowledge gaps were identified regarding vector recognition and transmission pathways. Misconceptions about mosquito-borne disease transmission are not uncommon and have been reported in studies assessing knowledge, attitudes, and practices regarding arboviral infections among healthcare students and communities [22]. These gaps may negatively influence risk communication and the ability of future healthcare professionals to provide effective preventive advice. Attitudes toward prevention were generally favorable. Most participants recognized the importance of community involvement, environmental control, and healthcare seeking symptomatic individuals. However, a discrepancy emerged between positive attitudes and perceived self-efficacy, as only a minority of students felt personally capable of preventing chikungunya. Similar findings have been reported in studies evaluating knowledge, risk perception, and preventive behaviors related to mosquito-borne arboviral diseases. In a recent survey among physicians in Germany, despite a generally high level of awareness regarding mosquito-borne arboviral diseases, differences were observed between perceived risk, knowledge, and the implementation of preventive practices, highlighting the importance of continuous professional education and targeted training [25]. Likewise, among international travelers, higher perceived risk of mosquito-borne infections was associated with greater adoption of personal protective behaviors, suggesting that risk perception plays a key role in translating awareness into effective prevention strategies [26]. Therefore, educational interventions targeting healthcare students should not only improve knowledge but also strengthen confidence and perceived ability to implement preventive measures. Improving self-efficacy may therefore represent an important objective of educational interventions. Preventive practices reported by participants were generally aligned with recommended vector-control strategies, particularly regarding removal of mosquito breeding sites, management of stagnant water, and environmental sanitation. However, because practices were self-reported, they may reflect general hygiene behaviors rather than actions specifically motivated by chikungunya awareness. Future studies should explore whether targeted education on chikungunya can translate into sustained disease-specific preventive behaviors. The findings of this study have important implications for public health and nursing education. The continuing expansion of *Aedes* mosquitoes, together with climate change and increased population mobility, may further increase the risk of arboviral disease emergence in Europe [15,16,17]. Nurses have a fundamental role in health promotion, patient education, early recognition of infectious diseases, and community engagement. Therefore, nursing curricula could benefit from more structured education on emerging vector-borne diseases, including basic medical entomology, recognition of relevant mosquito vectors, understanding of vector–pathogen transmission mechanisms, clinical recognition, and evidence-based preventive measures [18,19]. From an educational perspective, basic medical entomology may be integrated into broader teaching on infectious diseases, public health, epidemiology, and health promotion, providing students with fundamental knowledge of vector identification, transmission mechanisms, and prevention. This educational approach may be particularly relevant in settings where vector-borne diseases are not routinely encountered in clinical practice. In the present study, the low proportion of aware students who reported being able to recognize the *Aedes* mosquito (n = 4) suggests that vector identification represents a potential educational gap that deserves further investigation. Previous studies have also highlighted the importance of knowledge regarding mosquito vectors and transmission mechanisms for effective prevention of arboviral diseases [21,22]. Integrating these topics into undergraduate nursing education could strengthen future healthcare professionals’ ability to provide accurate health education and risk communication and to support community-based vector-control measures. Several limitations should be considered when interpreting the findings. First, the cross-sectional design precludes causal inference and limits the ability to determine temporal relationships between participant characteristics and awareness. Second, the study was conducted at a single university, the University of Palermo, and therefore the findings may not be generalizable to nursing students attending other Italian universities or to students enrolled in other healthcare disciplines. Differences in curricula, educational exposure to emerging infectious diseases, and local epidemiological contexts may influence awareness and knowledge of chikungunya. Future multicenter studies involving nursing students from universities located in northern, central, and southern Italy would provide a broader assessment of chikungunya awareness and KAP across different geographical and educational settings. Multicenter studies involving institutions from other European countries could further determine whether the findings observed in this study are consistent across different healthcare education systems and epidemiological contexts. In particular, the characteristics of the study population and the local educational and epidemiological context may differ across Italian regions. Multicenter studies involving nursing students from universities located in northern, central, and southern Italy, as well as studies including institutions from other European countries, would be useful to assess the consistency of these findings across different geographical and educational settings. Third, previous awareness of chikungunya was reported by only 22 participants, resulting in limited statistical power for subgroup analyses and wide confidence intervals for the prevalence ratio estimates. Therefore, the multivariable analysis should be considered exploratory, and the observed associations should be interpreted cautiously. Fourth, the use of self-reported data may have introduced recall bias and social desirability bias. An additional limitation concerns the questionnaire used for data collection. The instrument was developed ad hoc by the authors and was not adapted from a previously validated questionnaire, nor was it subjected to formal psychometric validation or structured pilot testing before administration. This limitation is particularly relevant for the KAP component, which was completed by only 22 participants who reported previous awareness of chikungunya. The small number of respondents precluded meaningful psychometric assessment of the attitude scale and limits the reliability and generalizability of the KAP findings. Therefore, the KAP results should be interpreted as exploratory and descriptive rather than as a validated measurement of chikungunya-related knowledge, attitudes, and practices. Future studies should use or develop validated instruments and should include expert assessment of content validity, pilot testing, and formal evaluation of reliability and construct validity before large-scale administration. Finally, because knowledge, attitudes, and practices were assessed only among participants who had heard of chikungunya, the corresponding findings should be interpreted cautiously. Despite these limitations, this study provides valuable preliminary evidence regarding chikungunya awareness among nursing students in Italy. Strengths include the relatively large sample size, the inclusion of students from different academic years, and the use of a structured questionnaire that allowed simultaneous assessment of awareness, knowledge, attitudes, and practices. To our knowledge, few studies have examined this topic in European nursing student populations, making the present findings a useful contribution to the literature on preparedness for emerging vector-borne diseases.

## 5. Conclusions

In conclusion, awareness of chikungunya among nursing students at the University of Palermo was extremely low despite the increasing relevance of arboviral diseases in Europe and the documented capacity of *Aedes albopictus* to sustain local transmission in Italy. Although students who were aware of chikungunya demonstrated generally positive attitudes and reported appropriate preventive practices, important knowledge gaps were identified, particularly regarding vector recognition, transmission mechanisms, and individual risk perception. These findings highlight the need to strengthen educational strategies targeting future healthcare professionals. From a public health perspective, the results suggest that preparedness for emerging vector-borne diseases should not rely exclusively on outbreak-driven responses but should be incorporated into long-term prevention strategies. For policymakers and public health authorities, integrating arboviral disease education into healthcare training programs represents an important opportunity to improve early recognition, risk communication, and community engagement. Nursing curricula could incorporate structured educational content on emerging vector-borne diseases, including chikungunya, dengue, and other mosquito-borne infections, with emphasis on basic medical entomology, vector identification, transmission mechanisms, epidemiology, clinical presentation, prevention, and vector-control measures. Such training could help future nurses provide accurate health education and risk communication and support community participation in vector-control activities. At the policy level, strengthening collaboration between universities, regional health authorities, and public health institutions may facilitate the development of coordinated training programs and surveillance activities. Healthcare students could represent an important component of public health preparedness by acting as future multipliers of accurate health information within communities. Educational interventions aimed at nursing students may therefore have a broader impact by improving public awareness and promoting preventive behaviors among the general population. Furthermore, considering the increasing influence of climate change, international mobility, and the expansion of *Aedes* mosquito populations in Europe, decision-makers should consider vector-borne disease preparedness as a priority within regional health planning. Investments in continuous professional education, community-based vector-control programs, and risk communication strategies may reduce the impact of future chikungunya outbreaks. In particular, southern European regions such as Sicily, where environmental conditions are favorable for mosquito proliferation, may benefit from targeted prevention strategies integrating surveillance, education, and community participation. Future public health policies should also encourage the development of standardized educational interventions and evaluation frameworks to monitor changes in knowledge, attitudes, and preventive behaviors among healthcare students. Given the single-center design of the present study, these findings should be considered preliminary and should be further evaluated through multicenter studies involving nursing students from different Italian regions and European settings. Strengthening the competencies of future nurses may represent a cost-effective approach to improving population resilience against emerging arboviral threats.

## Figures and Tables

**Table 1:** Sociodemographic characteristics of the study sample (n = 894).

Variable	Category	n	%
Have you ever heard of Chikungunya?	Yes	22	2.46
	No	872	97.54
Gender	Female	646	72.26
	Male	248	27.74
Year of study	First year	343	38.37
	Second year	280	31.32
	Third year	271	30.31
Country of birth	Italy	879	98.32
	Other country	15	1.68
Age (years)	Mean ± SD	22.33 ± 4.53	—

SD = standard deviation.

**Table 2:** Association between participants’ characteristics and awareness of Chikungunya (n = 894).

Variable	Category	No, n (%)	Yes, n (%)	*p*-Value
Gender	Female	631 (97.68)	15 (2.32)	0.665
	Male	241 (97.18)	7 (2.82)	
Year of study	First year	339 (98.83)	4 (1.17)	**0.011**
	Second year	275 (98.21)	5 (1.79)	
	Third year	258 (95.20)	13 (4.80)	
Country of birth	Italy	858 (97.61)	21 (2.39)	0.289
	Other country	14 (93.33)	1 (6.67)	
Total sample		872 (97.54)	22 (2.46)	-

Note: Data are presented as n (%). Pearson’s chi-square test was used to assess associations between participant characteristics and awareness of Chikungunya. Statistically significant *p*-values (*p* < 0.05) are shown in bold.

**Table 3:** Prevalence ratios for previous awareness of Chikungunya.

Variable	PR (95% CI)	*p*-Value
Gender (reference: female)		
Male	1.17 (0.47–2.93)	0.730
Age (per 1-year increase)	1.07 (1.00–1.15)	0.062
Country of birth (reference: Italy)		
Other	1.55 (0.11–21.87)	0.746
Year of study (reference: first year)		
Second year	1.45 (0.41–5.21)	0.565
Third year	3.38 (1.10–10.36)	**0.033**

Data are presented as adjusted prevalence ratios (PRs) with corresponding 95% confidence intervals (CIs) estimated using multivariable Poisson regression with robust variance. The dependent variable was previous awareness of Chikungunya (“having heard of Chikungunya”). Reference categories are indicated where applicable. Statistically significant *p*-values (*p* < 0.05) are shown in bold.

**Table 4:** Sources of Chikungunya-related information among participants who reported prior awareness of the disease (n = 22).

Source of Information	n	%
Mass media (television, newspapers, and other news media) ^a^	14	63.64
University lectures ^b^	5	22.73
Peers/colleagues ^c^	2	9.09
Yellow fever vaccination center	1	4.55
Previous health-related training	1	4.55
Familiarity with the disease in the country of origin	1	4.55
Mass media (television, newspapers, and other news media) ^a^	14	63.64

Notes: Data are presented as frequency (n) and percentage (%). Percentages do not sum to 100% because participants could report more than one source of information. ^a^ Includes responses indicating “Mass media (television/newspapers)” (n = 12), “Mass media (television/newspapers);” (n = 1), and the multiple-response category “University lectures; Mass media; Peers/colleagues” (n = 1). ^b^ Includes responses indicating “University lectures” (n = 4) and the multiple-response category “University lectures; Mass media; Peers/colleagues” (n = 1). ^c^ Includes responses indicating “Peers/colleagues” (n = 1) and the multiple-response category “University lectures; Mass media; Peers/colleagues” (n = 1).

**Table 5:** Knowledge, attitudes, and practices (KAPs) regarding Chikungunya (CHIK) among participants. Based on responses from 22 participants.

Domain	Item	Response Distribution	Mean Score
Knowledge	K1. Is Chikungunya (CHIK) caused by a virus?	**Yes: 17**; Don’t know: 3; No: 3	–
	K2. Can all mosquitoes transmit CHIK?	**No: 18**; Don’t know: 3; Yes: 1	–
	K3. Can you recognize the *Aedes* mosquito?	**Yes: 4**; No: 18	–
	K3bis. If yes, when is the *Aedes* mosquito most likely to feed/bite? (n = 10)	In any period: 6; At night: 3; **During the day: 1**	–
	K4. Do flies transmit CHIK?	**No: 13**; Don’t know: 8; Yes: 1	–
	K5. Do ticks transmit CHIK?	**No: 12**; Don’t know: 10	–
	K6. Can CHIK be transmitted through person-to-person contact?	Don’t know: 11; **No: 10**; Yes: 1	–
	K7. Is CHIK transmitted through food and water?	**No: 11**; Don’t know: 9; Yes: 2	–
	K8. Do mosquitoes breed in stagnant water?	**Yes: 17**; Don’t know: 4; No: 1	–
	K9. Do mosquito nets and bed nets reduce mosquitoes?	**Yes: 15**; Don’t know: 4; No: 3	–
	K10. Do insecticide sprays reduce mosquitoes and prevent CHIK?	**Yes: 9**; Don’t know: 9; No: 4	–
	K11. Does properly covering water containers reduce mosquitoes?	**Yes: 11**; Don’t know: 8; No: 3	–
	K12. Do mosquito repellents prevent mosquito bites?	**Yes: 16**; No: 3; Don’t know: 3	–
	K13. Does CHIK affect newborns, children, and adults?	**Yes: 20**; Don’t know: 2	–
	K14. What are the main signs and symptoms of CHIK?		
	K14a. Fever	**Yes: 21**; No: 1	–
	K14b. Joint pain	**Yes: 18**; No: 4	–
	K14c. Headache	**Yes: 19**; No: 3	–
	K14d. Muscle pain	**Yes: 21**; No: 1	–
	K14e. Bone pain	**Yes: 14**; No: 8	–
	K14f. Nausea	**Yes: 15**; No: 7	–
	K14g. Skin rash	**Yes: 16**; No: 6	–
Attitudes	A1. CHIK is a severe/life-threatening disease	Disagree (1–3): 15; **Agree (4–5): 7**	3.09
	A2. CHIK is preventable	Disagree (1–3): 10; **Agree (4–5): 12**	3.73
	A3. Water around homes may serve as breeding sites for *Aedes* mosquitoes	Disagree (1–3): 9; **Agree (4–5): 13**	3.91
	A4. Controlling breeding sites is a good strategy to prevent CHIK	Disagree (1–3): 7; **Agree (4–5): 15**	4.23
	A5. Communities should actively participate in vector control	Disagree (1–3): 5; **Agree (4–5): 17**	4.09
	A6. Everyone is at risk of CHIK infection	Disagree (1–3): 5; **Agree (4–5): 17**	4.00
	A7. Individuals with CHIK symptoms should immediately seek healthcare	Disagree (1–3): 5; **Agree (4–5): 17**	4.27
	A8. Everyone plays an important role in preventing CHIK	Disagree (1–3): 5; **Agree (4–5): 17**	4.18
	A9. All CHIK patients have the possibility of complete recovery	Disagree (1–3): 11; **Agree (4–5): 11**	3.45
	A10. I am capable of preventing CHIK	Disagree (1–3): 15; **Agree (4–5): 7**	3.14
	A11. I have a responsibility to prevent mosquito larvae around my home	Disagree (1–3): 10; **Agree (4–5): 12**	3.77
Practices	P1. Drain ponds or reclaim muddy/wet areas to reduce mosquitoes	**Yes: 19**; No: 3	–
	P2. Keep bushes short to reduce mosquitoes	No: 12; **Yes: 10**	–
	P3. Clean waste/garbage to reduce mosquito breeding sites	**Yes: 18**; No: 4	–
	P4. Dispose of water-holding containers (tires, plastics, bottles, broken pots)	**Yes: 20**; No: 2	–
	P5. Use a fan or air conditioning to prevent mosquito bites	No: 16; **Yes: 6**	–
	P6. Wear protective clothing when working in bush, rural, or forested areas	**Yes: 18**; No: 4	–
	P7. Cover water storage containers at home	**Yes: 17**; No: 5	–
	P8. Change water in household plant containers weekly	**Yes: 19**; No: 3	–
	P9. Control waste that may block water flow around the house	**Yes: 18**; No: 4	–
	P10. Frequently clean water-filled containers and household drains	**Yes: 20**; No: 2	–

Note: Correct knowledge responses, positive attitudes, and recommended preventive practices are shown in bold. Attitude items were assessed using a 5-point Likert scale (1 = strongly disagree; 5 = strongly agree).

## Data Availability

The raw data supporting the conclusions of this article will be made available by the authors on request.
